# 2-[(1*S*,3*S*)-3-Acetyl-2,2-dimethyl­cyclo­butyl]-*N*-(*m*-tol­yl)acetamide

**DOI:** 10.1107/S160053680706641X

**Published:** 2007-12-18

**Authors:** Yan-Bai Yin, Zhan-Qian Song, Zong-De Wang, Shi-Bin Shang

**Affiliations:** a Institute of Chemical Industry of Forest Products, Chinese Academy of Forestry, Nanjing 210042, People’s Republic of China; b College of Forestry, Jiangxi Agricultural University, Nanchang 330045, People’s Republic of China

## Abstract

The title compound, C_17_H_23_NO_2_, contains two chiral centres and was synthesized from 2-(3-acetyl-2,2-dimethyl­cyclo­butyl)acetic acid and *m*-toluidine. The cyclobutane ring is not flat but flexed as though folded from the dimethyl-substituted C atom to the unsubstituted C atom, with a dihedral angle of 25.9°. The crystal structure is stabilized by N—H⋯O and C—H⋯O hydrogen-bonding inter­actions.

## Related literature

For related literature, see: Mitra & Khanra (1977 ▶); Yin *et al.* (2007 ▶).
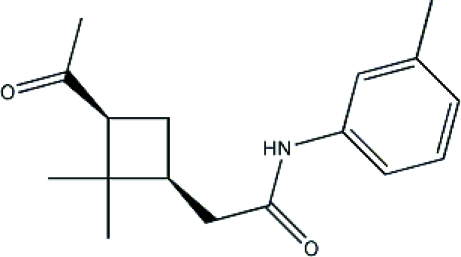


## Experimental

### 

#### Crystal data

C_17_H_23_NO_2_
*M* *_r_* = 273.36
Orthorhombic, 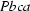

*a* = 12.513 (3) Å
*b* = 9.5190 (19) Å
*c* = 26.844 (5) Å
*V* = 3197.4 (11) Å^3^
*Z* = 8
Mo *K*α radiation
μ = 0.07 mm^−1^
*T* = 293 (2) K
0.40 × 0.20 × 0.20 mm

#### Data collection

Enraf–Nonius CAD-4 diffractometer
Absorption correction: ψ scan (North *et al.*, 1968 ▶) *T*_min_ = 0.951, *T*_max_ = 0.975
3150 measured reflections
3120 independent reflections
1385 reflections with *I* > 2σ(*I*)
*R*_int_ = 0.032
3 standard reflections every 200 reflections intensity decay: none

#### Refinement

*R*[*F* ^2^ > 2σ(*F* ^2^)] = 0.073
*wR*(*F* ^2^) = 0.170
*S* = 1.04
3120 reflections
181 parameters
H-atom parameters constrained
Δρ_max_ = 0.14 e Å^−3^
Δρ_min_ = −0.14 e Å^−3^

### 

Data collection: *CAD-4 Software* (Enraf–Nonius, 1989 ▶); cell refinement: *CAD-4 Software*; data reduction: *XCAD4* (Harms & Wocadlo, 1995 ▶); program(s) used to solve structure: *SHELXS97* (Sheldrick, 1997*a* ▶); program(s) used to refine structure: *SHELXL97* (Sheldrick, 1997*a* ▶); molecular graphics: *SHELXTL* (Sheldrick, 1997*b* ▶); software used to prepare material for publication: *SHELXTL*.

## Supplementary Material

Crystal structure: contains datablocks I, global. DOI: 10.1107/S160053680706641X/at2522sup1.cif

Structure factors: contains datablocks I. DOI: 10.1107/S160053680706641X/at2522Isup2.hkl

Additional supplementary materials:  crystallographic information; 3D view; checkCIF report

## Figures and Tables

**Table 1:** Hydrogen-bond geometry (Å, °)

*D*—H⋯*A*	*D*—H	H⋯*A*	*D*⋯*A*	*D*—H⋯*A*
N—H0*A*⋯O2^i^	0.86	2.04	2.892 (4)	169
C12—H12*A*⋯O2	0.93	2.49	2.931 (5)	109
C13—H13*A*⋯O1^ii^	0.93	2.55	3.440 (5)	161

Symmetry codes: (i) 
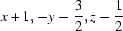
; (ii) 
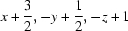
.

**Fig. 1.**
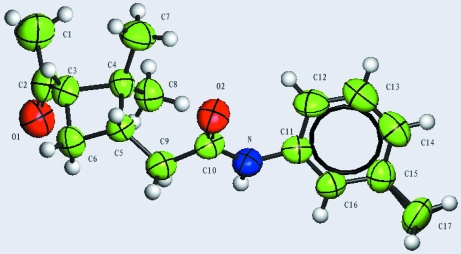
A view of the molecular structure of (I), showing displacement ellipsoids at the 50% probability level.

**Fig. 2.**
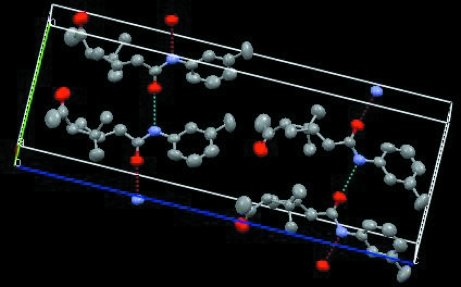
A view of the packing and N—H···O hydrogen bondings (dash lines) of the title compound.

## supplementary crystallographic information

### Comment

Terpenes are convenient chiral precursors due to their availability and low cost, and among them, a-pinene (both enantiomers) and verbenone are prominent. For instance, pinene has been used as starting material for the production of some compounds of industrial interest (Mitra & Khanra, 1977). Chiral cyclobutane compound, pinonic acid, can be synthesized from a-pinene. Many derivatives of pinonic acid have interesting biological properties. So we synthesized several derivatives of pinonic acid. In our previous paper we have reported the crystal structure of 2-[(1*S*,3S)-3-acetyl-2,2-dimethylcyclobutyl]-*N*-(2,6-difluorophenyl) acetamide (Yin *et al.*, 2007). Now we synthesized the title compound (I) and report here its crystal structure.

The molecular structure of (I) is shown in Fig. 1. A l l bond lengths and angles are normal. The crystal structure is stabilized by N—H···O and C—H···O hydrogen bonding interactions (Table 1).

### Experimental

The title compound was synthesized from *m*-toluidine and 2-(3-acetyl-2,2-dimethylcyclobutyl) acetyl chloride at room temperature. The acetyl chloride was obtained using 2-(3-acetyl-2,2-dimethylcyclobutyl)acetic acid (pinonic acid), thionyl chloride as raw materials and dichloromethane as solvent. Pinonic acid (27 mmol) and thionyl chloride (32 mmol) were dissolved in dichloromethane (50 ml). The resulting mixture was refluxed for 8 h. After refluxing the solvent was distilled away under vacuum and the remainder was 2-(3-acetyl-2,2-dimethylcyclobutyl)acetyl chloride. The acetyl chloride reacted with *m*-toluidine (27 mmol) for 24 h using dichloromethane as solvent. After the reaction was complete the solvent was distilled away and the crude title compound was gained. The pure compound was obtained by crystallizing from a mixture of ethanol (40 ml) and water (40 ml). Crystals of the title compound suitable for X-ray diffraction were obtained by slow evaporation of an ethanol solution.

### Refinement

All H atoms were placed geometrically, with the C—H distances in the range 0.93–0.98 Å and N—H = 0.86 Å, and included in the refinement in riding motion approximation with *U*_iso_(H) = 1.2 or 1.5*U*_eq_(H) of the carrier atom.

### Crystal data

**Table:**

C_17_H_23_NO_2_	*D*_x_ = 1.136 Mg m^−^^3^
*M**_r_* = 273.36	Melting point: 367 K
Orthorhombic, *P**b**c**a*	Mo *K*α radiation λ = 0.71073 Å
Hall symbol: -P 2ac 2ab	Cell parameters from 25 reflections
*a* = 12.513 (3) Å	θ = 8–13º
*b* = 9.5190 (19) Å	µ = 0.07 mm^−^^1^
*c* = 26.844 (5) Å	*T* = 293 (2) K
*V* = 3197.4 (11) Å^3^	Quadrate, colourless
*Z* = 8	0.40 × 0.20 × 0.20 mm
*F*_000_ = 1184	

### Data collection

**Table:**

Enraf–Nonius CAD-4 diffractometer	*R*_int_ = 0.032
Radiation source: fine-focus sealed tube	θ_max_ = 26.0º
Monochromator: graphite	θ_min_ = 1.5º
*T* = 293(2) K	*h* = 0→15
ω/2θ scans	*k* = 0→11
Absorption correction: ψ scan(North *et al.*, 1968)	*l* = 0→32
*T*_min_ = 0.951, *T*_max_ = 0.975	3 standard reflections
3150 measured reflections	every 200 reflections
3120 independent reflections	intensity decay: none
1385 reflections with *I* > 2σ(*I*)	

### Refinement

**Table:**

Refinement on *F*^2^	Secondary atom site location: difference Fourier map
Least-squares matrix: full	Hydrogen site location: inferred from neighbouring sites
*R*[*F*^2^ > 2σ(*F*^2^)] = 0.073	H-atom parameters constrained
*wR*(*F*^2^) = 0.170	*w* = 1/[σ^2^(*F*_o_^2^) + (0.05*P*)^2^] where *P* = (*F*_o_^2^ + 2*F*_c_^2^)/3
*S* = 1.04	(Δ/σ)_max_ < 0.001
3120 reflections	Δρ_max_ = 0.14 e Å^−^^3^
181 parameters	Δρ_min_ = −0.14 e Å^−^^3^
Primary atom site location: structure-invariant direct methods	Extinction correction: none

### Special details

**Table:**

Geometry. All e.s.d.'s (except the e.s.d. in the dihedral angle between two l.s. planes) are estimated using the full covariance matrix. The cell e.s.d.'s are taken into account individually in the estimation of e.s.d.'s in distances, angles and torsion angles; correlations between e.s.d.'s in cell parameters are only used when they are defined by crystal symmetry. An approximate (isotropic) treatment of cell e.s.d.'s is used for estimating e.s.d.'s involving l.s. planes.
Refinement. Refinement of *F*^2^ against ALL reflections. The weighted *R*-factor *wR* and goodness of fit *S* are based on *F*^2^, conventional *R*-factors *R* are based on *F*, with *F* set to zero for negative *F*^2^. The threshold expression of *F*^2^ > σ(*F*^2^) is used only for calculating *R*-factors(gt) *etc*. and is not relevant to the choice of reflections for refinement. *R*-factors based on *F*^2^ are statistically about twice as large as those based on *F*, and *R*- factors based on ALL data will be even larger.

### Fractional atomic coordinates and isotropic or equivalent isotropic displacement parameters (Å^2^)

**Table:**

	*x*	*y*	*z*	*U*_iso_*/*U*_eq_	
N	0.7095 (2)	0.0658 (3)	0.68825 (10)	0.0721 (8)	
H0A	0.7277	−0.0171	0.6977	0.087*	
O1	0.6178 (2)	−0.0407 (3)	0.92753 (10)	0.1064 (9)	
C1	0.4701 (3)	0.1175 (5)	0.93668 (16)	0.1272 (16)	
H1A	0.4414	0.0449	0.9576	0.191*	
H1B	0.4227	0.1333	0.9091	0.191*	
H1C	0.4774	0.2026	0.9556	0.191*	
O2	0.7021 (2)	0.2940 (2)	0.71316 (9)	0.0883 (8)	
C2	0.5782 (3)	0.0728 (4)	0.91735 (14)	0.0830 (11)	
C3	0.6333 (3)	0.1758 (3)	0.88408 (12)	0.0684 (9)	
H3A	0.6270	0.2708	0.8978	0.082*	
C4	0.5977 (3)	0.1749 (3)	0.82785 (11)	0.0643 (9)	
C5	0.7192 (2)	0.1983 (3)	0.81526 (11)	0.0642 (8)	
H5A	0.7331	0.2993	0.8128	0.077*	
C6	0.7475 (3)	0.1488 (4)	0.86728 (11)	0.0777 (10)	
H6A	0.7682	0.0507	0.8688	0.093*	
H6B	0.7999	0.2077	0.8838	0.093*	
C7	0.5230 (3)	0.2938 (4)	0.81276 (14)	0.1015 (13)	
H7A	0.4510	0.2698	0.8216	0.152*	
H7B	0.5274	0.3081	0.7774	0.152*	
H7C	0.5435	0.3784	0.8297	0.152*	
C8	0.5565 (3)	0.0333 (3)	0.81108 (12)	0.0782 (10)	
H8A	0.4824	0.0246	0.8197	0.117*	
H8B	0.5966	−0.0397	0.8272	0.117*	
H8C	0.5645	0.0249	0.7756	0.117*	
C9	0.7689 (3)	0.1250 (3)	0.77045 (12)	0.0771 (10)	
H9A	0.8452	0.1427	0.7705	0.092*	
H9B	0.7584	0.0245	0.7738	0.092*	
C10	0.7236 (3)	0.1716 (3)	0.72154 (12)	0.0662 (9)	
C11	0.6677 (3)	0.0789 (3)	0.63926 (13)	0.0650 (9)	
C12	0.5888 (3)	0.1753 (3)	0.62739 (16)	0.0855 (11)	
H12A	0.5602	0.2353	0.6513	0.103*	
C13	0.5545 (3)	0.1783 (4)	0.57870 (19)	0.1012 (13)	
H13A	0.5033	0.2443	0.5698	0.121*	
C14	0.5914 (3)	0.0904 (4)	0.54333 (16)	0.0931 (12)	
H14A	0.5657	0.0978	0.5109	0.112*	
C15	0.6686 (3)	−0.0127 (4)	0.55487 (14)	0.0801 (10)	
C16	0.7049 (3)	−0.0140 (3)	0.60417 (13)	0.0723 (9)	
H16A	0.7559	−0.0800	0.6135	0.087*	
C17	0.7134 (3)	−0.1128 (5)	0.51691 (14)	0.1146 (14)	
H17A	0.6788	−0.0977	0.4854	0.172*	
H17B	0.7011	−0.2077	0.5276	0.172*	
H17C	0.7888	−0.0971	0.5134	0.172*	

### Atomic displacement parameters (Å^2^)

**Table:**

	*U*^11^	*U*^22^	*U*^33^	*U*^12^	*U*^13^	*U*^23^
N	0.088 (2)	0.0515 (15)	0.077 (2)	0.0067 (14)	0.0022 (16)	0.0046 (15)
O1	0.115 (2)	0.099 (2)	0.105 (2)	−0.0141 (18)	−0.0003 (17)	0.0199 (17)
C1	0.085 (3)	0.175 (4)	0.122 (4)	−0.020 (3)	0.026 (3)	−0.006 (3)
O2	0.124 (2)	0.0526 (14)	0.0881 (17)	−0.0010 (13)	0.0038 (15)	0.0082 (12)
C2	0.102 (3)	0.079 (2)	0.068 (2)	−0.015 (2)	−0.003 (2)	−0.007 (2)
C3	0.078 (2)	0.0583 (18)	0.069 (2)	−0.0117 (18)	0.0023 (18)	−0.0043 (17)
C4	0.074 (2)	0.0616 (19)	0.057 (2)	−0.0020 (17)	−0.0052 (17)	0.0061 (16)
C5	0.063 (2)	0.0605 (19)	0.070 (2)	−0.0078 (16)	0.0058 (17)	0.0026 (17)
C6	0.067 (2)	0.092 (2)	0.074 (2)	−0.0174 (18)	−0.0040 (19)	0.006 (2)
C7	0.097 (3)	0.098 (3)	0.110 (3)	0.025 (2)	0.002 (2)	0.015 (2)
C8	0.077 (2)	0.081 (2)	0.077 (2)	−0.0201 (19)	−0.0042 (19)	0.0009 (19)
C9	0.080 (2)	0.072 (2)	0.079 (2)	0.0042 (18)	0.008 (2)	0.0053 (19)
C10	0.078 (2)	0.0522 (19)	0.069 (2)	0.0044 (18)	0.0147 (18)	0.0129 (19)
C11	0.067 (2)	0.0499 (18)	0.079 (2)	−0.0047 (16)	0.0074 (19)	0.0102 (19)
C12	0.084 (3)	0.059 (2)	0.113 (3)	0.013 (2)	0.001 (2)	−0.003 (2)
C13	0.090 (3)	0.096 (3)	0.117 (4)	0.002 (3)	−0.022 (3)	0.011 (3)
C14	0.088 (3)	0.090 (3)	0.101 (3)	−0.015 (2)	−0.025 (2)	0.014 (3)
C15	0.085 (3)	0.085 (3)	0.071 (3)	−0.012 (2)	0.003 (2)	0.000 (2)
C16	0.074 (2)	0.068 (2)	0.075 (2)	0.0018 (19)	0.012 (2)	0.006 (2)
C17	0.109 (3)	0.153 (4)	0.081 (3)	0.001 (3)	0.017 (2)	−0.036 (3)

### Geometric parameters (Å, °)

**Table:**

N—C10	1.357 (4)	C7—H7B	0.9600
N—C11	1.421 (4)	C7—H7C	0.9600
N—H0A	0.8600	C8—H8A	0.9600
O1—C2	1.219 (4)	C8—H8B	0.9600
C1—C2	1.510 (5)	C8—H8C	0.9600
C1—H1A	0.9600	C9—C10	1.497 (4)
C1—H1B	0.9600	C9—H9A	0.9700
C1—H1C	0.9600	C9—H9B	0.9700
O2—C10	1.217 (3)	C11—C16	1.374 (4)
C2—C3	1.495 (4)	C11—C12	1.384 (4)
C3—C6	1.521 (4)	C12—C13	1.376 (5)
C3—C4	1.574 (4)	C12—H12A	0.9300
C3—H3A	0.9800	C13—C14	1.347 (5)
C4—C8	1.512 (4)	C13—H13A	0.9300
C4—C7	1.522 (4)	C14—C15	1.411 (5)
C4—C5	1.573 (4)	C14—H14A	0.9300
C5—C6	1.516 (4)	C15—C16	1.399 (4)
C5—C9	1.523 (4)	C15—C17	1.504 (5)
C5—H5A	0.9800	C16—H16A	0.9300
C6—H6A	0.9700	C17—H17A	0.9600
C6—H6B	0.9700	C17—H17B	0.9600
C7—H7A	0.9600	C17—H17C	0.9600
			
C10—N—C11	126.3 (3)	H7B—C7—H7C	109.5
C10—N—H0A	116.9	C4—C8—H8A	109.5
C11—N—H0A	116.9	C4—C8—H8B	109.5
C2—C1—H1A	109.5	H8A—C8—H8B	109.5
C2—C1—H1B	109.5	C4—C8—H8C	109.5
H1A—C1—H1B	109.5	H8A—C8—H8C	109.5
C2—C1—H1C	109.5	H8B—C8—H8C	109.5
H1A—C1—H1C	109.5	C10—C9—C5	113.7 (3)
H1B—C1—H1C	109.5	C10—C9—H9A	108.8
O1—C2—C3	121.8 (4)	C5—C9—H9A	108.8
O1—C2—C1	122.5 (4)	C10—C9—H9B	108.8
C3—C2—C1	115.7 (4)	C5—C9—H9B	108.8
C2—C3—C6	120.0 (3)	H9A—C9—H9B	107.7
C2—C3—C4	116.1 (3)	O2—C10—N	124.0 (3)
C6—C3—C4	88.9 (2)	O2—C10—C9	121.9 (3)
C2—C3—H3A	110.1	N—C10—C9	114.0 (3)
C6—C3—H3A	110.1	C16—C11—C12	120.7 (4)
C4—C3—H3A	110.1	C16—C11—N	116.9 (3)
C8—C4—C7	112.0 (3)	C12—C11—N	122.3 (3)
C8—C4—C3	112.7 (3)	C13—C12—C11	117.0 (4)
C7—C4—C3	115.2 (3)	C13—C12—H12A	121.5
C8—C4—C5	113.0 (3)	C11—C12—H12A	121.5
C7—C4—C5	115.5 (3)	C14—C13—C12	123.4 (4)
C3—C4—C5	86.1 (2)	C14—C13—H13A	118.3
C6—C5—C9	119.3 (3)	C12—C13—H13A	118.3
C6—C5—C4	89.1 (2)	C13—C14—C15	120.8 (4)
C9—C5—C4	120.0 (3)	C13—C14—H14A	119.6
C6—C5—H5A	108.9	C15—C14—H14A	119.6
C9—C5—H5A	108.9	C16—C15—C14	115.9 (3)
C4—C5—H5A	108.9	C16—C15—C17	120.9 (4)
C3—C6—C5	90.0 (2)	C14—C15—C17	123.2 (4)
C3—C6—H6A	113.6	C11—C16—C15	122.2 (3)
C5—C6—H6A	113.6	C11—C16—H16A	118.9
C3—C6—H6B	113.6	C15—C16—H16A	118.9
C5—C6—H6B	113.6	C15—C17—H17A	109.5
H6A—C6—H6B	110.9	C15—C17—H17B	109.5
C4—C7—H7A	109.5	H17A—C17—H17B	109.5
C4—C7—H7B	109.5	C15—C17—H17C	109.5
H7A—C7—H7B	109.5	H17A—C17—H17C	109.5
C4—C7—H7C	109.5	H17B—C17—H17C	109.5
H7A—C7—H7C	109.5		
			
O1—C2—C3—C6	−7.8 (5)	C4—C5—C6—C3	−18.5 (2)
C1—C2—C3—C6	173.1 (3)	C6—C5—C9—C10	172.5 (3)
O1—C2—C3—C4	97.1 (4)	C4—C5—C9—C10	64.8 (4)
C1—C2—C3—C4	−82.0 (4)	C11—N—C10—O2	−0.5 (5)
C2—C3—C4—C8	−27.7 (4)	C11—N—C10—C9	179.7 (3)
C6—C3—C4—C8	95.5 (3)	C5—C9—C10—O2	40.0 (5)
C2—C3—C4—C7	102.6 (4)	C5—C9—C10—N	−140.2 (3)
C6—C3—C4—C7	−134.3 (3)	C10—N—C11—C16	149.2 (3)
C2—C3—C4—C5	−141.0 (3)	C10—N—C11—C12	−34.2 (5)
C6—C3—C4—C5	−17.8 (2)	C16—C11—C12—C13	−3.5 (5)
C8—C4—C5—C6	−95.2 (3)	N—C11—C12—C13	−180.0 (3)
C7—C4—C5—C6	134.0 (3)	C11—C12—C13—C14	2.1 (6)
C3—C4—C5—C6	17.9 (2)	C12—C13—C14—C15	0.5 (6)
C8—C4—C5—C9	28.6 (4)	C13—C14—C15—C16	−1.6 (5)
C7—C4—C5—C9	−102.2 (3)	C13—C14—C15—C17	−179.5 (4)
C3—C4—C5—C9	141.7 (3)	C12—C11—C16—C15	2.5 (5)
C2—C3—C6—C5	138.2 (3)	N—C11—C16—C15	179.1 (3)
C4—C3—C6—C5	18.5 (2)	C14—C15—C16—C11	0.2 (5)
C9—C5—C6—C3	−142.9 (3)	C17—C15—C16—C11	178.1 (3)

### Hydrogen-bond geometry (Å, °)

**Table:**

*D*—H···*A*	*D*—H	H···*A*	*D*···*A*	*D*—H···*A*
N—H0A···O2^i^	0.86	2.04	2.892 (4)	169
C12—H12A···O2	0.93	2.49	2.931 (5)	109
C13—H13A···O1^ii^	0.93	2.55	3.440 (5)	161

Symmetry codes: (i) *x*+1, −*y*−3/2, *z*−1/2; (ii) *x*+3/2, −*y*+1/2, −*z*+1.
